# Research on Active Safety Situation of Road Passenger Transportation Enterprises: Evaluation, Prediction, and Analysis

**DOI:** 10.3390/e26060434

**Published:** 2024-05-21

**Authors:** Lili Zheng, Shiyu Cao, Tongqiang Ding, Jian Tian, Jinghang Sun

**Affiliations:** 1 Transportation College, Jilin University, Changchun 130022, China; lilizheng@jlu.edu.cn (L.Z.); caosy22@mails.jlu.edu.cn (S.C.); jhsun22@mails.jlu.edu.cn (J.S.); 2 China Academy of Transportation Sciences, Beijing 100029, China; tianjian@motcats.ac.cn

**Keywords:** transportation enterprises, active safety situation, factor analysis, time series, model selection, statistical computing, feature selection, statistical inference

## Abstract

The road passenger transportation enterprise is a complex system, requiring a clear understanding of their active safety situation (ASS), trends, and influencing factors. This facilitates transportation authorities to promptly receive signals and take effective measures. Through exploratory factor analysis and confirmatory factor analysis, we delved into potential factors for evaluating ASS and extracted an ASS index. To predict obtaining a higher ASS information rate, we compared multiple time series models, including GRU (gated recurrent unit), LSTM (long short-term memory), ARIMA, Prophet, Conv_LSTM, and TCN (temporal convolutional network). This paper proposed the WDA-DBN (water drop algorithm-Deep Belief Network) model and employed DEEPSHAP to identify factors with higher ASS information content. TCN and GRU performed well in the prediction. Compared to the other models, WDA-DBN exhibited the best performance in terms of MSE and MAE. Overall, deep learning models outperform econometric models in terms of information processing. The total time spent processing alarms positively influences ASS, while variables such as fatigue driving occurrences, abnormal driving occurrences, and nighttime driving alarm occurrences have a negative impact on ASS.

## 1. Introduction

Road transportation vehicles carry a large number of passengers, and when a traffic accident occurs, it often results in more serious casualties and property damage. On 19 July 2021, a passenger bus collided with a heavy vehicle in Pakistan’s eastern province of Punjab, resulting in at least 27 deaths and more than 30 injuries. On 12 September 2020, on the 24th of the Mecklenburg—formerly Federal State of Pomerania in northern Germany—highway, a Hamburg-bound passenger bus suddenly left the highway and entered a ditch, injuring 31 people [1]. On 28 September 2019, at about 7:00 p.m., a particularly major road traffic accident in which a bus collided with a heavy semi-trailer automobile train occurred on the Jiangsu Wuxi section of China’s Changshen Expressway, resulting in 36 deaths, 36 injuries, and more than CNY 71 million in direct economic losses [2].

In order to effectively reduce accidents, enterprises need to implement comprehensive safety measures such as employee transportation safety and operational procedure training; establish safety management principles and guidelines; deploy advanced monitoring systems; and utilize advanced driver assistance systems and other technological equipment for accident warning and prevention. Efforts have been made globally to enhance the safety of road passenger transportation enterprises. The Ministry of Transport of the People’s Republic of China has issued safety management regulations for road passenger transportation enterprises, detailing the requirements for driver recruitment conditions, pre-job training, safety education and training, assessment, driving and rest time, and long-distance shuttle transportation. It is also recommended that each province develop and operate dynamic monitoring platforms for vehicles to monitor fatigue alarms, overspeed alarms, and alarms for abnormal drivers. Directive 2006/126/EC of the European Union specifies the physical health conditions required for driver qualification certificates, Directive 2003/59/EC stipulates the training requirements for professional truck and bus drivers, and the European Agreement Concerning the Work of Crews of Vehicles Engaged in International Road Transport (AETR) is used to prevent drivers of certain commercial vehicles engaged in international road transport from exceeding specified driving times. Compliance with working and rest time regulations is recorded on digital tachographs, which can provide information on any violations of regulations. The US Department of Transportation has established many safety regulations for transportation enterprises, such as requiring the employment of drivers holding valid commercial driver’s licenses and conducting alcohol and drug testing, the regular inspection and maintenance of operating vehicles, and prohibiting drivers from driving severely overtime [3]. The specific requirements for ensuring transportation safety vary among these countries. For example, regarding working hours and rest time: In China, passenger drivers are limited to a maximum of 4 h of continuous driving during the day and 2 h at night, with a minimum rest period of 20 min for each stop, and the cumulative driving time within 24 h shall not exceed 8 h, and shall not exceed 44 h within any consecutive 7 days. In the European Union, the maximum continuous driving time is limited to 4.5 h, with a minimum rest period of 45 min for each stop, and the cumulative driving time within 24 h shall not exceed 9 h, and shall not exceed 56 h within any consecutive week. In the United States, drivers may drive a maximum of 10 h after resting for 8 consecutive hours, and may not drive after working for 60/70 h over 7/8 consecutive days. Several countries rely on platforms or onboard devices for monitoring driving and rest time regulations, and also detect and warn against more dangerous driving behaviors. These rich data provide important support for assessing the current situation and trends of transportation enterprise safety and taking proactive measures to prevent accidents from occurring.

In fact, for individual drivers, the occurrence of accidents is extremely fortuitous; for enterprises, the number and severity of accidents show a certain distribution pattern. Therefore, if noise and redundancy can be eliminated from the information during transmission, and the dynamic changes in the safety of each enterprise can be effectively tracked, targeted feedback can be provided to take regulatory measures, which will help prevent accidents more effectively and ensure the safety of road passenger transportation enterprises.

Some researchers directly use accidents, collisions, or their probabilities to assess traffic safety. Traffic accidents are often a direct reflection of traffic safety, but traffic accident data are often difficult to obtain, especially in developing countries [4,5]. Moreover, when no accident occurs, the safety is actually not exactly the same, and it is difficult to accurately describe the safety when no accident occurs if only accident data is relied upon. Ding et al. [6] found a large correlation between the occurrence of safety violations and traffic accidents. Rahman et al. [7] found that fatigue can have a large impact on traffic safety. Similar to the speed change, TTC (time to collision), DRAC (deceleration rate to avoid collision) [8], TET (collision exposure time indicator), and other alternative indicators of safety are used in micro collision research [9], and it is possible to select or construct alternative indicators of the macro traffic safety situation of enterprises from them. They are aimed at solving the problem of the difficult-to-obtain data of traffic accidents on the basis of different indicators instead of traffic accidents. By utilizing alternative safety assessment methods that do not rely on traffic accident data, transportation managers and planners can gain a better understanding of the safety performance of transportation systems.

The road transportation enterprise is a complex system composed of drivers, vehicles, safety management personnel, dynamic monitoring systems, and business management. They intertwine in time and space, collectively influencing the overall safety operation of the system. In order to dynamically assess the situation of the enterprise, this paper draws on the idea of alternative safety assessment and proposes the concept of active safety situation (ASS). ASS is a macroscopic description of the safety situation of road passenger transportation enterprises that does not rely on accident information. It reflects the complex safety situation and evolutionary trends of the enterprise, specifically referring to the entire system’s safety situation and change trends influenced by interactions among drivers, vehicles, safety management personnel, dynamic monitoring systems, and business management elements. Furthermore, the active safety situation index (ASSI) is used to quantify ASS. Enterprises and transportation management departments can utilize time series forecasting models to predict future trends based on historical ASS. And, they can analyze the impact of driver feedback, equipment utilization, and dynamic monitoring intensity on ASS, and control factors that have a negative impact on ASS. Based on the results, transportation authorities can gain an insight into the future trends of the industry in advance. And by understanding the factors affecting the safety of the industry, it can formulate more scientific and reasonable traffic safety planning decisions to improve the safety of road passenger transportation enterprises and reduce the incidence of traffic accidents. For enterprises, it can assess the reasonableness and safety of the dynamic management to optimize the operation strategy and improve the operation safety.

The rest of this paper is divided into four parts. A literature review is described in Section 2. Section 3 describes the sources of the data and the methods used. Section 4 describes the experimental setup and results. Section 5 describes the discussion. Finally, Section 6 gives the conclusion of this paper and the future research direction.

## 2. Literature Review

### 2.1. Assessment of the Safety Situation of Enterprises

For public safety reasons, various countries conduct road safety assessments, which are aimed at countries, provinces, cities, states, and so on. Direct assessment indicators commonly used in these assessments are injury severity, the number of casualties, relative accident risk, the occurrence of accidents, or the probability of accidents in traffic accident data. Zhang et al. [10] used injury severity for each year from 2006 to 2010. The expected number of injuries in potential crashes was used [11]. Zhang et al. [12] used the incidence and severity of injuries and fatalities in crashes for each year between 2006 and 2010. Malin et al. [13] used the relative crash risk for single-vehicle and multi-vehicle crashes between 2014 and 2016. Parsa et al. [14] used XGBoost to assess the occurrence or non-occurrence of accidents through 244 traffic accidents and 6073 non-accident cases from December 2016 to December 2017.

The safety assessment of traffic passenger transportation enterprises often focuses on management aspects, overlooking driver operation characteristics. Dong et al. [15] applied accident causation theory and system safety engineering theory to develop a factor structure affecting enterprise safety. They constructed a multi-level safety assessment index system considering enterprise qualification management, transportation organization, employee factors, and accidents. Similarly, Wu et al. [16] developed a first-level multi-objective decision-making model using dynamic multi-value background and entropy theory. They introduced a relative entropy-based quadratic assembly optimization model for group decision making, tailored specifically for dangerous goods transportation enterprises, based on cluster analysis principles and relative entropy theory.

The above study primarily focuses on long-term assessment. While annual and monthly data offer valuable insights into enterprise safety, they lack the granularity needed for daily assessment due to their lower temporal resolution. As the application of monitoring platforms spreads, more daily data can be obtained, which provides the possibility of analyzing the ASS of an enterprise by the day. To enhance traffic safety management comprehensively and accurately, practical needs necessitate daily-scale assessments. Such assessments will empower managers to promptly respond to changes in safety situations, allowing for more flexible safety strategies and ultimately improving enterprise safety.

There is also a desire for a comprehensive assessment indicator to judge the long-term safety of enterprises. In Europe, a comprehensive assessment is carried out regarding the enterprise’s compliance with regulations, inspections, and safety standards. In the United States, the safety of long-distance highway passenger carriers is assessed by the Federal Motor Carrier Safety Administration (FMCSA). FMCSA is a part of the U.S. Department of Transportation, and uses a safety measurement system to rate carriers on the basis of their compliance with safety regulations and the results of interventions. China uses the Implementing Rules for Standardized Evaluation of Enterprise Safety Production to assess the safety of enterprises.

Currently, researchers believe that there is a correlation between the occurrence of safety violations and traffic accidents, and that more safety situations lead to a higher probability of traffic accidents, and the number of violations is the most critical variable affecting the accident propensity of bus drivers [17]. This provides new ideas for the construction of a dynamic ASS of enterprises.

### 2.2. Application of Time Series Forecasting to Traffic Safety

Prediction involves analyzing historical information to forecast the most likely future occurrences of information. In the past studies on long-term forecasting of traffic safety situations, researchers usually used time series analysis to describe, explain, and predict the general trend of traffic safety situations. Time series analysis is widely used in the field of road transportation and road safety, especially in the study of traffic accidents. However, we note that the traffic safety situation is a dynamic process, and most of the existing macro-evaluations of traffic safety rely on annual or monthly data on traffic accidents.

Given the current challenge of long-forecasting time horizons, we need methods to understand daily trend changes in enterprise ASS more accurately. Prediction methods are essentially categorized into two types: causal-based and time series-based predictions. Causal-based forecasting is often difficult to predict future situations in advance. Therefore, for advance insight, time series-based forecasting is preferred. Many researchers believe that there is a serial correlation in the occurrence of accidents or injuries, so they use the number of accidents as a time series and explore its seasonality [18]. Antoniou and Yannis [19] used a risk time series (LRT) model to predict the number of deaths per year in Greece for 52 years (1960–2011). Yousefzadeh-Chabok et al. [20] used the SARIMA (1, 1, 3) (0, 1, 0)12 model to predict the road traffic accident fatality rate in Zanjan Province, Iran, from 2007 to 2013. Getahun [21] used the ARIMA model to predict the number of accidents per month in the Amhara region, Ethiopia. Rabbani et al. [22] used the Seasonal Autoregressive Integrated Moving Average (SARIMA) and Exponential Smoothing (ES) models to predict monthly accident rates in Pakistan. Barba et al. [23] addressed time series smoothing by preprocessing data with three-point moving average smoothing or the singular value decomposition of Hankel matrix (HSVD) pairs. They employed an ARIMA model and two ANN to predict weekly traffic accident injuries in Chile’s Valparaiso region from 2003 to 2012. Bao et al. [24] proposed a spatial–temporal convolutional short-term memory network (STCL-Net) comprising CNN, LSTM, and Conv_LSTM components for weekly and daily collision risk prediction in cities. To construct a daily crash number prediction model for 2020 crash forecasts across the United States, de Zarza et al. [25] used Transformer, ARIMA, and Prophet. Commandeur et al. [26] used the DRAG model and ARIMA to forecast annual national road traffic fatalities. Uguz and Buyukgokoglan [27] applied SARIMA, Prophet, LSTM, and a proposed hybrid CNN-LSTM to predict daily traffic accident frequency during the tourist season in Antalya.

Econometric models and deep learning models are both widely utilized for forecasting time series data. Each of them possesses distinct advantages and applicability in information extraction and mining from time series data. Researchers should select a specific model based on data characteristics and problem complexity.

### 2.3. Driver Operational Characteristics Affecting Traffic Safety

Humans, vehicles, roads, and the environment all play important roles in traffic safety. However, human characteristics are often the most difficult to control and predict compared to the other characteristics. And, most accidents occur due to human errors [28]. The conditions of the vehicles have an impact on traffic safety. Mechanical failures, broken parts, or vehicles that do not meet safety standards can increase the risk of accidents. But, regular vehicle maintenance and inspection can dramatically reduce the risks posed by vehicles. As road characteristics are studied, safer roads can be designed over time, and the relatively static nature of road characteristics allows risky road sections to be prevented in advance.

Researchers found that driver violations while driving could have an impact on safety conditions. Bucsuházy et al. and Yaman et al. [28,29] found that driver negligence such as distracted state, distraction, overloaded driving, and seat belt use had an impact on traffic safety. Moradi et al. [30] concluded that fatigued driving had an impact on traffic safety. Useche et al. [31] proved that fatigue and work stress had a significant impact on the working conditions of long-distance transportation drivers. Doecke et al. [32] found a positive exponential relationship between speed limits and fatal crash rates. Therefore, many researchers have built on this knowledge and made further studies on fatigue driving and speed limits to improve the impact of speed limits on highway traffic safety [33]. Choudhary et al. [34] argued that drivers underestimated the risks associated with phone conversations. Driver sleepiness due to circadian rhythm disruption and sleep restriction was also a non-negligible cause of accidents [35]. Zeller et al. [36] demonstrated that both sleep demand and task duration negatively affected driver status and that task duration reduces driver performance in the absence of sleep demand. For these reasons, many countries have implemented nighttime travel bans. Currently, there is evidence that nighttime travel bans may be an effective way to reduce the burden of road traffic crashes and road traffic fatalities in Zambia and other low- and middle-income countries [37].

Due to advancements in information collection and transmission technologies, the monitoring and warning of these dangerous behaviors are centralized on the platform. When a driver violation alarm occurs, the driver must address and provide feedback on the violation. However, researchers have yet to explore the necessity of the bidirectional transmission of this information, as factors like equipment usage and driver feedback have not been studied for their impact on safety. Additionally, the platform gathers data such as driving distance, the number of operating vehicles, and equipment utilization rates. The relationship between these data and enterprises’ ASS requires further investigation.

### 2.4. Research Gap and Contribution

Enterprises require safety assessments and predictions, not only monthly, quarterly, and annual, but also daily. Real-time awareness is critical to reflect the impact of dynamic traffic safety management measures and industry regulations in a timely manner. By integrating continuous safety monitoring into daily operations, enterprises can proactively identify and respond to emerging safety challenges, thereby creating a safer and more resilient transportation environment.

This paper identifies two major research gaps in assessing and predicting the ASS of road passenger transportation enterprises:

- Most of the above studies have assessed and predicted traffic accident data and the general scope of the study is a country. It is possible for a country to generate traffic accidents on a daily basis, so safety has been studied yearly, monthly, weekly, and daily. However, road passenger transportation enterprises do not have traffic accidents most of the time, so it is difficult to assess and predict their safety dynamically through traffic accident data on a daily basis;
- The impact of driver feedback violation alarms and equipment usage on safety has not yet been studied.

The following three steps will be used in this paper to fill these research gaps:

1. Considering alarms, driver feedback, and equipment usage, an ASSI is constructed using exploratory factor analysis and validation factor analysis methods to obtain the ASS of each enterprise;
2. Relying on the ASSI, we predict the future trend of the enterprises’ ASS based on time series model;
3. The WDA-DBN model is proposed, and the deep SHAP method is borrowed to dig deeper into the multifaceted variables that have an impact on ASS.

**Figure 1:**
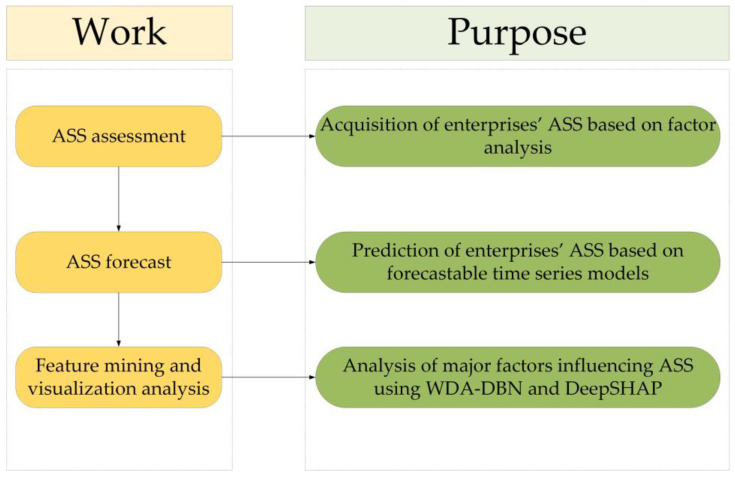
Framework.

**Figure 2:**
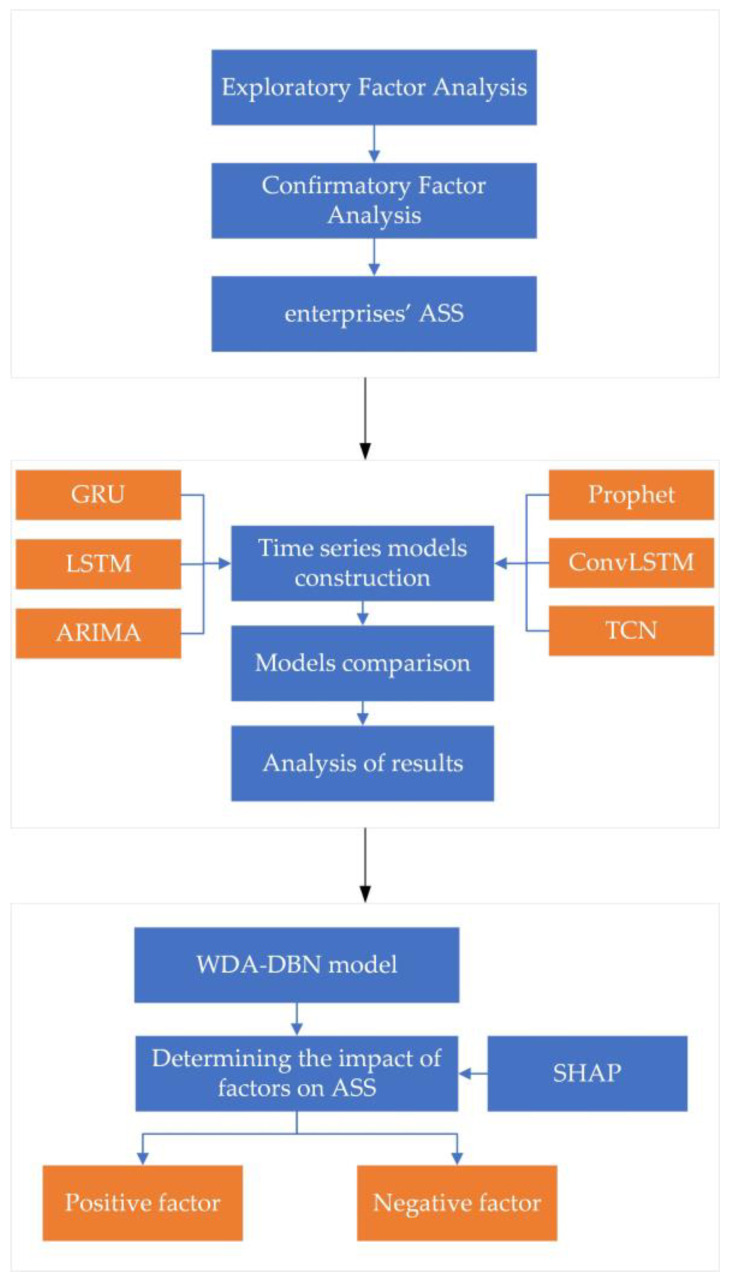
Framework details.

In this paper, we will use the technical route illustrated in Figure 1 and Figure 2 to conduct an in-depth study from the three perspectives mentioned above.

## 3. Methodology

### 3.1. Data

This study utilized data from the road transport vehicle supervision platform of a certain province in China, involving 296 road passenger transportation enterprises. The specific data range spans from 1 March 2022, to 26 May 2023, covering a total of 452 days. The detectors used include video detectors, Beidou positioning devices, and other equipment. For alarm-type information, management personnel carefully review the video recordings before uploading to the platform to verify and validate the alarm information. This process ensures the accuracy and reliability of the data. Enterprise information is directly entered into the backend. Data such as satellite positioning mileage and speed are transmitted directly to the platform by Beidou positioning devices. Since researchers believe that alarms are closely related to accidents [38], we integrated six types of alarm class datasets, namely, night ban, speeding, fatigue driving, equipment usage, alarm response, and the number of alarms, by using the same enterprise names and dates to obtain all the study data as shown in Table 1.

The missing data need to be processed as there is missing information on some of the variables in the original six datasets. The missing data are categorized into two types, one for some enterprises with all the data missing, and one for some enterprises with some days of data missing for individual variables. Since the missing data in the study are all the data of 2 road passenger transportation enterprises for 452 days, the data of these 2 enterprises are deleted, leaving 294 road passenger transportation enterprises, and the sample size becomes 294×452=132,888. For a few days of random missing data of individual variables in some enterprises, because the data is time series data, the linear interpolation method is used to estimate the missing values according to the linear relationship between the observed values before and after.

Since different variables tend to have different scales and scale units, and the drivers who drive illegally in the enterprise are a minority, the variables do not satisfy the normal distribution; so, in order to facilitate the subsequent experiments, the variables are now subjected to Z-Score standardization. To ensure that the value of each index is non-negative, on the basis of Z-Score standardization, another Min–max normalization is performed. The variables of the data set of road transportation enterprises are shown in Table 1.

### 3.2. ASS Assessment Methodology Based on Factor Analysis

The data introduced in Section 3.1 has a variety of variables. To accurately dig out the key variables that can reflect the ASS of the enterprise, this thesis adopted the factor analysis. Starting with the dependency relationship within the correlation matrix of the research variables, we attributed some variables with overlapping information and intricate relationships together and dug out the factors with potential relationships that can represent the ASS of the enterprise in a large amount of information. Two types of factor analysis methods were utilized: exploratory factor analysis and confirmatory factor analysis.

#### 3.2.1. Exploratory Factor Analysis

Exploratory factor analysis (EFA) is an a priori theory-free method of revealing the underlying relationships and structure between observed variables. Its goal is identifying the number and structure of potential common factors and revealing the associations between each observed variable and these factors. When a variable has a high value of loadings with a factor, it means that the variable is strongly correlated with that factor, which implies that the factor explains the variable more significantly. Respectively, factor loading and factor variance are shown as

(1) $$\Phi =\left[\begin{array}{cccc} \lambda_{11} & \lambda_{12} & \cdots & \lambda_{1k}\\ \lambda_{21} & \lambda_{22} & \cdots & \lambda_{2k}\\ \vdots & \vdots & \ddots & \vdots \\ \lambda_{p1} & \lambda_{p2} & \cdots & \lambda_{pk} \end{array}\right]$$

(2) $$\Psi =\left[\begin{array}{cccc} \Psi_{1}^{2} & 0 & \cdots & 0\\ 0 & \Psi_{2}^{2} & \cdots & 0\\ \vdots & \vdots & \ddots & \vdots \\ 0 & 0 & \cdots & \Psi_{k}^{2} \end{array}\right]$$

where $\lambda_{ij}$ is the factor loading between observed variable i and latent factor j, p is the number of variables, k denotes the number of latent factors, and $\Psi_{j}^{2}$ is the variance of latent factor j. The variance of latent factor *j* was calculated by using the following formula:

(3) $$\Psi_{j}^{2}=\sum_{i=1}^{p}\lambda_{ij}^{2}$$

The main steps of EFA include determining the number of factors, extracting factors, rotating factors, and interpreting factors. In this paper, the number of factors is determined according to the criterion that the characteristic root is greater than 1 [39].

To verify the validity of the exploration, Kaiser–Meyer–Olkin (KMO) and Bartlett’s sphericity test were required.

KMO measure is a statistical indicator used in EFA to assess the suitability of the sample data, which measures the suitability of the correlation between the variables for factor analysis. The value of KMO lies between 0 and 1, and the closer it is to 1 indicates that the sample is more suitable for the factor analysis. The KMO value is calculated by the formula shown in Equation (4):

(4) $$KMO=\frac{\sum \sum_{i\neq j}r_{ij}^{2}}{\sum \sum_{i\neq j}r_{ij}^{2}+\sum \sum_{i\neq j}\tau_{ij}^{2}}$$

where $r_{ij}^{2}$ is the square of the correlation coefficient between variable i and variable j, and $\tau_{ij}^{2}$ is the square of the partial correlation coefficient between variable i and variable j. A KMO value greater than 0.6 is generally considered acceptable for EFA.

Before conducting factor analysis, Bartlett’s sphericity test is typically performed to ensure that the selected set of features is suitable for factor analysis. The null hypothesis of this test is that there is no correlation between features, meaning that the covariance matrix of features is a multiple of the identity matrix, exhibiting sphericity. If the *p*-value of Bartlett’s sphericity test is lower than the predetermined significance level (usually 0.05), the null hypothesis is rejected, indicating the presence of correlations between features and suitability for factor analysis. Conversely, if the *p*-value is higher, the null hypothesis cannot be rejected, implying a lack of correlation between features, and thus unsuitability for factor analysis.

#### 3.2.2. Confirmatory Factor Analysis

Confirmatory factor analysis (CFA) is a data dimensionality reduction method with a priori theory. It aims to verify whether a predefined factor structure matches the data. If the hypothesized factor structure matches the actual data, the hypothesized factor structure is acceptable. The measured variables within the factor can be screened with factor loading coefficients, which are calculated as shown in Equation (4):

(5) $$\beta_{ij}=\sqrt{\tau_{j}}\lambda_{ij}$$

where $\beta_{ij}$ denotes the factor loadings between the observed variable i and the latent factor j, $\tau_{j}$ denotes the variance of the latent factor j, and $\lambda_{ij}$ denotes the standardized factor loadings. Generally, a standardized loading coefficient value greater than 0.6 can indicate that the measured variable meets the factor requirements.

The average variance extraction AVE is used to measure the internal consistency of the construct, i.e., the proportion of the variance of the variable explained by the construct to the total variance of the construct. For construct i, AVE is calculated as follows:

(6) $${AVE}_{i}=\frac{\sum_{j=1}^{k}\lambda_{ij}^{2}}{\sum_{j=1}^{k}\lambda_{ij}^{2}+\sum_{j=1}^{k}\Psi_{ij}^{2}}$$

where $\lambda_{ij}$ is the standardized loading of the *j*th indicator in construct i and $\Psi_{ij}$ is the error variance of the jth indicator in construct i. If the AVE is greater than or equal to 0.5, it means that the variance of the variable explained by the construct occupies a relatively large portion of the total variance, and that the construct has better explanatory power.

The combined reliability CR is used to assess the reliability of the construct, i.e., the consistency of the indicators within the construct [40]. For conceptualization i, CR is calculated as follows:

(7) $${CR}_{i}=\frac{{\left(\sum_{j=1}^{k}\lambda_{ij}\right)}^{2}}{{\left(\sum_{j=1}^{k}\lambda_{ij}\right)}^{2}+\sum_{j=1}^{k}\Psi_{ij}^{2}}$$

If the CR is greater than or equal to 0.7, it means that there is a high degree of consistency between the indicators within the conceptualization, and the reliability of the conceptualization is good.

### 3.3. A Time Series-Based Method for Predicting ASS

Enterprise safety situation is one of the crucial considerations in the management and decision making of today’s organizations. With the rapid development of technology, more and more types and quantities of data can be collected. Enterprises and transportation authorities can properly use these data to improve the safety of enterprises, which requires the real-time monitoring and prediction of the enterprises’ ASS. Time series analysis plays a key role in this context, providing a powerful tool to understand and respond to changing trends in ASS.

Time series data, such as alarm and driver feedback, have unique properties such as trend, seasonality, and periodicity that introduce challenges and complexity to enterprises’ ASS analysis. Nassiri et al. [41] believed that traffic safety had seasonality, so they used SARIMAX with explaining variables, the univariate autoregressive differential moving average model, and the seasonal time series model to make autoregressive predictions on the monthly total number of accidents and fatal accidents on all rural roads in Iran. Similarly, threats and safety incidents faced by enterprises may exhibit gradual trends, seasonal effects, or clear cycles. Therefore, selecting the appropriate time series analysis method is essential for accurately capturing these patterns and predicting future trends in enterprise safety.

In this study, we explored and compared multiple time series analysis methods to identify a model that can better extract the active safety posture information rate in the active safety posture system of road transportation enterprises, with the goal of minimizing distortion. We specifically selected gated recurrent units, long- and short-term memory networks, autoregressive moving average models, prophet models, convolutional long- and short-term memory networks, and temporal convolutional network methods [42,43,44,45] based on their unique advantages in dealing with enterprise safety data with complex dynamics. Through a comprehensive comparison of these approaches, we aimed to provide powerful tools and methods for enterprise safety management to better cope with enterprise unsafety.

#### 3.3.1. Gated Recurrent Unit (GRU)

The gated recurrent unit (GRU) is a variable of Recurrent neural network (RNN) designed to solve the problem of gradient vanishing and gradient explosion in traditional RNNs. GRU is simplified from long short-term memory by introducing two gating mechanisms to control the updating and output of the hidden state without introducing an additional memory unit.

The hidden state of GRU is controlled by two gating units: the update gate and the reset gate. The update gate determines whether the hidden state is updated to the input of the current time step. The reset gate determines how to combine the previous hidden state and the input of the current time step to compute the new hidden state. The input to the update gate is the ASSI of the current time step and the hidden state of the previous time step. The output is the retained hidden state of the previous time step. The reset gate input is the ASSI of the current time step t and the hidden state of the previous time step. The output is whether to reset the hidden state of the previous time step. These gating mechanisms are unique in preserving long-term ASSI information without discarding it over time, as it is unrelated to prediction [46]. The formulas for updating gates, resetting gates, and hidden states are shown in Equations (8)–(11):

(8) $$z_{t}=\sigma \left(W_{z}\left[h_{t-1},x_{t}\right]\right)$$

(9) $$r_{t}=\sigma \left(W_{r}\left[h_{t-1},x_{t}\right]\right)$$

(10) $$h_{t}=\left(1-z_{t}\right)⊙h_{t-1}+z_{t}⊙\tilde{h_{t}}$$

(11) $$\tilde{h_{t}}=\tanh\left(W_{h}\left[r_{t}⊙h_{t-1},x_{t}\right]\right)$$

where $W_{z}$, $W_{r}$, and $W_{h}$ are the weight matrices, σ is the sigmoid activation function, $\left[h_{t-1},x_{t}\right]$ denotes the linking of the hidden states to the inputs, $x_{t}$ is the input of the input sequence at time step t, $h_{t-1}$ is the hidden state at the previous time step, tanh is the hyperbolic tangent activation function, ⊙ denotes the element-by-element multiplication, $z_{t}$ denotes the output of the update gate, $r_{t}$ denotes the output of the reset gate, and $\tilde{h_{t}}$ is the output of the new candidate value.

#### 3.3.2. Long Short-Term Memory Network (LSTM)

Long short-term memory (LSTM) is a variable of RNN. LSTM adds the mechanism of gates, which can control the flow of information, including input gates, forget gates, and output gates. The input of the input gate is the ASSI of the current time step and the hidden state of the previous time step, and the output is the extent to which new information should be added to the candidate’s hidden state. The forget gate input is the ASSI of the current time step and the hidden state of the previous time step, and the output is the extent to which the hidden state of the previous time step is to be forgotten. The output gate input is the ASSI of the current time step, the hidden state of the previous time step, and the cell state of the current time step, and the output is the degree of output of the hidden state of the current time step [47]. The formulas for the input gate, forgetting gate, output gate, and hidden state are shown:

(12) $$i_{t}=\sigma \left(W_{i}\left[h_{t-1},x_{t}\right]\right)$$

(13) $$f_{t}=\sigma \left(W_{f}\left[h_{t-1},x_{t}\right]\right)$$

(14) $$o_{t}=\sigma \left(W_{o}\left[h_{t-1},x_{t}\right]\right)$$

(15) $$h_{t}=o_{t}⊙tanh(C_{t})$$

(16) $$C_{t}=f_{t}⊙C_{t-1}+i_{t}⊙\tilde{C_{t}}$$

(17) $$\tilde{C_{t}}=tanh\left(W_{c}\left[h_{t-1},x_{t}\right]\right)$$

where $W_{i}$, $W_{f}$, $W_{o}$, and $W_{c}$ are the weight matrices, $C_{t}$ is the update of the memory cell, σ is the sigmoid activation function, $\left[h_{t-1},x_{t}\right]$ denotes connecting the hidden states to the inputs, $x_{t}$ is the input of the input sequence at time step t, $h_{t-1}$ is the hidden state of the previous time step, and $h_{t}$ is the hidden state of the current time step. tanh is the hyperbolic tangent activation function, ⊙ denotes element-by-element multiplication, $i_{t}$ denotes the output of the input gate, $f_{t}$ denotes the output of the forgetting gate, $o_{t}$ denotes the output of the output gate, and $\tilde{C_{t}}$ the output of the new candidate value.

#### 3.3.3. Autoregressive Integrated Moving Average (ARIMA)

Autoregressive Integrated Moving Average (ARIMA) is a model established by transforming data into smooth data through differencing, and then regressing the dependent variable only on its lagged value and the present and lagged values of the random error term. In ARIMA(*p*,*d*,*q*), AR is autoregressive, p is the number of autoregressive terms; MA is sliding average, *q* is the number of sliding average terms; *I* is the number of sliding average terms; *d* is the number of terms to make the autoregressive model a more efficient one; and d is the number of sliding average terms to make the autoregressive model a more efficient one. The autoregressive part represents the linear relationship between the observations at the current moment and the observations at several past moments [48]. The autoregressive part, the difference part, and the sliding average part of the ARIMA model can be represented as Equations (18)–(20), respectively:

(18) $$AR\left(P\right):X_{t}=c+\emptyset_{1}X_{t-1}+\emptyset_{2}X_{t-2}+\ldots +\emptyset_{p}X_{t-p}+\varepsilon$$

(19) $$I\left(d\right):Y_{t}=X_{t}-X_{t-d}$$

(20) $$MA\left(q\right):X_{t}=c+\varepsilon_{t}+\theta_{1}\varepsilon_{t-1}+\theta_{2}\varepsilon_{t-2}+\ldots +\theta_{q}\varepsilon_{t-q}$$

where $X_{t}$ is the ASSI at the current moment, $\emptyset_{1}$, $\emptyset_{2}$, ⋯, $\emptyset_{p}$ are autoregressive coefficients, c is a constant term, $\varepsilon_{t}$ is the white noise error, $Y_{t}$ is the time series after differencing, and $\theta_{1}$, $\theta_{2}$, ⋯, $\theta_{q}$ are moving average coefficients.

Ultimately, the prediction equation for the ARIMA model is Equation (21):

(21) $${\hat{X}}_{t+h}=c+\emptyset_{1}X_{t+h-1}+\emptyset_{2}X_{t+h-2}+\ldots +\emptyset_{p}X_{t+h-p}+\theta_{1}\varepsilon_{t+h-1}+\theta_{2}\varepsilon_{t+h-2}+\ldots +\theta_{q}\varepsilon_{t+h-q}$$

where ${\hat{X}}_{t+h}$ is the predicted value at future moment t+h.

#### 3.3.4. Prophet

Prophet model is a time series predicting model based on the additivity model, which decomposes the time series into four main components: trend, seasonality, holiday effect, and noise. Among them, trend is the long-term change trend of the time series, seasonality is the pattern of cyclical changes, holiday effect refers to the influence of special dates or time periods, and noise is the random changes that cannot be predicted. The time series decomposition stage of the Prophet model consists of two parts: trend decomposition and seasonality decomposition. The trend decomposition is modeled using a segmented linear function that divides the trend into linear and nonlinear components. The seasonal decomposition is modeled using a Fourier series that divides the seasonality into multiple cycles. In the parameter learning stage, the Prophet model uses least squares to estimate the regression coefficients [49]. The trend model, seasonality model, and holiday effect can be represented as Equations (22)–(24), respectively:

(22) $$g\left(t\right)=k+at+\sum_{i=1}^{G}C_{i}sigmoid(t-t_{i})$$

(23) $$s\left(t\right)=\sum_{n=1}^{N}\left(a_{n}\sin\left(\frac{2\pi nt}{P}\right)+b_{n}\cos\left(\frac{2\pi nt}{P}\right)\right)$$

(24) $$h\left(t\right)=\sum_{j}K_{j}I$$

where k is an offset term indicating the overall average, a is the slope of the linear trend, t is time, G is the number of inflection points in the nonlinear trend, $C_{i}$ is the growth rate at each inflection point $t_{i}$, N is the order of the seasonal pattern, P is the period of the seasonality, and $K_{j}$ is the effect associated with the jth holiday. I is the indicator function, which is 1 if the time t is in the jth holiday and 0 otherwise.

Finally, Prophet can be expressed as Equation (25):

(25) $$y\left(t\right)=g\left(t\right)+s\left(t\right)+h\left(t\right)+\varepsilon_{t}$$

where $y\left(t\right)$ is the ASSI at time *t*, $g\left(t\right)$ is the trend component, $s\left(t\right)$ is the seasonal component, $h\left(t\right)$ is the holiday effect, and $\varepsilon_{t}$ is the error term.

#### 3.3.5. Convolutional Long Short-Term Memory Networks (Conv_LSTM)

Convolutional long short-term memory network (Conv_LSTM) is a deep learning model that combines a convolutional neural network (CNN) and LSTM. The basic structure of Conv_LSTM is that a convolutional operation is added to the recurrent structure of LSTM. When computing the hidden state, Conv_LSTM performs a convolution operation on the input and the hidden state of the previous time step. There are input gates, forgetting gates, and output gates in Conv_LSTM. The input gate inputs are the ASSI of the current time step, the hidden state of the previous time step, and the memory cells of the current time step, and the output is the degree of updating the memory cells. The input to the forget gate is the ASSI of the current time step, the hidden state of the previous time step, and the memory cells of the current time step, and the output is the degree of forgetting the memory cells of the previous time step. The output gate inputs are the ASSI of the current time step, the hidden state of the previous time step, and the memory cells of the current time step, and the output is the output of the hidden state of the current time step [50]. The state updating and hidden state computation formulas of the Conv_LSTM are shown in Equations (26)–(31):

(26) $$i_{t}=\sigma \left(W_{i}*\left[h_{t-1},x_{t}\right]+b_{i}\right)$$

(27) $$f_{t}=\sigma \left(W_{f}*\left[h_{t-1},x_{t}\right]+b_{f}\right)$$

(28) $$o_{t}=\sigma \left(W_{o}*\left[h_{t-1},x_{t}\right]+b_{o}\right)$$

(29) $$h_{t}=o_{t}⊙tanh(C_{t})$$

(30) $$C_{t}=f_{t}⊙C_{t-1}+i_{t}⊙\tilde{C_{t}}$$

(31) $$\tilde{C_{t}}=tanh\left(W_{c}*\left[h_{t-1},x_{t}\right]+b_{c}\right)$$

where ∗ denotes the convolution operation, $b_{f}$ is the bias term for the forget gate, $b_{i}$ is the bias term for the input gate, $b_{o}$ is the bias term for the output gate, and $b_{c}$ is the bias term for the new candidate cell state.

#### 3.3.6. Temporal Convolutional Network (TCN)

Temporal convolutional network (TCN) utilizes a one-dimensional convolutional neural network to process time series data. The network structure of TCN is mainly composed of one-dimensional convolutional layers and residual blocks. Among them, the one-dimensional convolutional layer is used to extract the features of the time series data, while the residual block is used to deepen the depth of the network and improve the performance of the model. In the residual block, a technique called “inflated convolution” is used to increase the sensory field without increasing the number of parameters, thus improving the performance of the model [51]. The formula for inflated convolution is as follows:

(32) $$F\left(S\right)=\sum_{i=0}^{k-1}f\left(i\right)x_{s-di}$$

where d is the expansion factor, k is the filter size, $f\left(i\right)$ is the weight of the convolution kernel, and s−di represents the past direction. d takes the values of 1, 2, 4, and 8 chosen according to the model performance, and k between 3, 4, and 5 chosen according to the model performance.

The computation of the residual block can be expressed as follows:

(33) $$\mathrm{output}=\mathrm{ReLU}\left(\mathrm{Conv}\left(\mathrm{input}\right)+\mathrm{input}\right)$$

where Conv is a one-dimensional convolutional layer and ReLU is a modified linear unit activation function.

Here, we use mean square error (MSE) and mean absolute error (MAE) for evaluating the prediction performance of the proposed model. The formulas are shown in Equations (34) and (35):

(34) $$\mathrm{MSE}=\frac{1}{n}\sum_{i=1}^{n}{\left(y_{i}-{\hat{y}}_{i}\right)}^{2}$$

(35) $$\mathrm{MAE}=\frac{1}{n}\sum_{i=1}^{n}\left|y_{i}-{\hat{y}}_{i}\right|$$

where n is the number of samples, $y_{i}$ is the true value, and ${\hat{y}}_{i}$ is the predicted value.

### 3.4. Feature Mining and Visualization Analysis Method Based on WDA-DBN

A variety of variables jointly affect the ASS of the enterprise. To accurately mine the relevant variables affecting the ASS of the enterprise, this thesis proposed the water drop algorithm-Deep Belief Network (WDA-DBN) method to mine some of the variables in 3.1 and compared them with the baseline models DBN, back propagation neural network (BPNN), and Extreme Gradient Boosting(XGBOOST) [52]. On the best effect model WDA-DBN, deep SHAP was used to mine the relationship of the specific influence of each variable on the ASS of the enterprise.

#### 3.4.1. Deep Belief Network (DBN)

Deep Belief Network (DBN) is a deep neural network model based on unsupervised learning and consists of multiple Restricted Boltzmann Machine (RBM).

RBM is an undirected graph model based on an energy model, consisting of a visible layer and a hidden layer. With no connection between the two layers and neuron states of 0 or 1, the weights can be any real numbers. Its training is performed by maximizing the likelihood function of the training data and using gradient descent to adjust the weights. Training consists of forward propagation (the input data is computed through the hidden layer to reconstruct the data) and backpropagation (the reconstructed data error is computed and the weights are adjusted). Since the RBM model is an energy-based model, it uses an energy function to compute the initial weights of its globally optimal network [53]. The energy function is represented as in Equation (36):

(36) $$E\left(V,H\right)=-\sum_{i=1}^{n}\sum_{j=1}^{m}w_{ij}v_{i}h_{j}-\sum_{i=1}^{n}a_{i}v_{i}-\sum_{j=1}^{m}b_{j}h_{j}$$

where $w_{ij}$ denotes the connection weight from the visible unit $v_{i}$ to the hidden unit $h_{j}$, n denotes the number of neurons in the visible layer, m denotes the number of neurons in the hidden layer, $a_{i}$ denotes the bias of the visible layer i, and $b_{j}$ denotes the bias of the hidden layer j. $w_{ij}$, $a_{i}$, and $b_{j}$ are real numbers representing the values of the connection weight, the visible bias, and the hidden bias, respectively.

Since there is no connection in a single layer, the conditional probabilities of the visible layer $v_{i}$ and the hidden unit $h_{j}$ are independent and can be computed as Equations (37) and (38):

(37) $$p\left(h_{j}=1|v\right)=s\left(\sum_{i=1}^{m}w_{ij}v_{j}+b_{j}\right)$$

(38) $$p\left(v_{i}=1|h\right)=s\left(\sum_{j=1}^{n}w_{ij}h_{j}+a_{i}\right)$$

where $s\left(x\right)$ is a sigmoid function with $s\left(x\right)=1/\left(1+exp\left(-x\right)\right)$.

The training process of DBN is divided into two phases: pre-training and fine-tuning. In the pre-training phase, each RBM is trained individually to learn the features of the filtered input data. In the fine-tuning phase, the entire DBN is trained as a supervised feedforward neural network to optimize the performance of the target task. In the pre-training phase, each RBM is trained as a self-encoder, i.e., the input data is encoded and decoded by the RBMs so that the reconstructed output layer can restore the input layer as much as possible while obtaining the dimensionality reduction results of the intermediate hidden layers. This process can be realized by the Contrastive Divergence (CD) algorithm. In the fine-tuning phase, the parameters of the DBN are adjusted to minimize the loss function for the target task, which is usually achieved using a backpropagation algorithm.

#### 3.4.2. WDA-DBN

In DBN, weights and bias parameters are key components of the network and are adjusted by the CD algorithm and backpropagation algorithm introduced in Section 3.4.1. Balancing the avoidance of overfitting while preserving or even enhancing the learning advantages brought by deep architectures remains a continual research topic in the field of deep learning [54]. When optimizing the weight parameters of the DBN regression model, we employ L2 regularization to implement the water drop algorithm (WDA), assisting the DBN model in exploring common patterns of information transmission as thoroughly as possible. The WDA mimics the sliding process of water droplets on terrain. Through multiple iterations, each water droplet gradually influences subsequent ones, facilitating information transmission and coordinated parameter adjustments. This analogy aids in optimizing the global search space and enhancing algorithm robustness. WDA dynamically adjusts parameter updating speed based on different problems and data, overcoming the limitations of traditional gradient descent methods. Specifically, WDA works by calculating the 2-norm (L2 norm) for each parameter and adding it as a regularization term to the loss function. The total loss function of the model is regarded as terrain height, which usually represents the degree of superiority and disadvantage in the search space. The L2 regular term of each parameter is regarded as the movement of water droplets. One water drop is used in each round, and multiple water droplets are used in multiple rounds, which gradually affect the following water droplets (in the traditional water drop algorithm, water droplets interact with each other). Here we let the front water drop affect the back water drop. This could limit the size of parameters, adjust the interlayer weight and threshold of the hidden layer of the neural network, avoid overfitting, and improve the generalization ability of the model. Using WDA can help DBN better fit the training data through backpropagation and optimizer updates. The loss function of WDA backpropagation is calculated as shown in Equation (39):

(39) $$Loss=loss+\sum_{k=1}^{T}{L2}_{k}lambd$$

where loss is the loss of the DBN model, ${L2}_{k}$ is the L2 regularization for the kth parameter, T is the number of parameters, and lambd is a fixed parameter taken as 0.001.

#### 3.4.3. SHAP

SHAP is a model-agnostic approach that derives its foundation from game theory. Its goal is to explain the prediction f(x) of an instance x by calculating the relative contribution of each feature value to a particular outcome. SHAP originates from the field of interpretable artificial intelligence and represents a form of Shapley values. Shapley values can be utilized in deep learning to quantify the contribution of each feature in model predictions, serving as a superior quantification of the information content of each feature. For features with higher information content, one can opt for compression algorithms with lower loss to ensure the integrity of information. The name of SHAP is defined as the prediction of a single instance. SHAP values can be approximated by different methods, e.g., kernel SHAP, deep SHAP, Tree SHAP, linear SHAP, etc. In this study, as DBN is a deep learning model, in order to identify the most important features while eliminating irrelevant, redundant, and noisy features, we employ DEEPSHAP to further explore the impact of various factors on ASS [55]. DeepLIFT is an attribution method for understanding the contribution of the model to the prediction of the input. In deep learning, the multipliers of DeepLIFT are redefined as SHAP values. By calculating the SHAP value for each feature in the deep learning model, it helps us to understand the impact of each feature on ASS. Deep SHAP combines the SHAP values calculated for smaller components in the network into a SHAP value for the entire network. It does this by recursively passing the DeepLIFT multiplier (now the SHAP value) backwards through the network.

The Shapley values of the features in the model are given by Equation (40):

(40) $$Shapley\left(x_{j}\right)=\sum_{S\subseteq N\backslash \left\{j\right\}}\frac{k!\left(p-k-1\right)!}{p!}(f(S\cup \left\{j\right\})-f(S))$$

where p is the total number of features, $N\backslash \left\{j\right\}$ is the set of all possible combinations of features except $x_{j}$, S is the set of features in $N\backslash \left\{j\right\}$, f(S) is the model prediction with features in S, and $f(S\cup \left\{j\right\})$ is the model prediction with features in S plus the feature $x_{j}$.

Here again, we used MSE and MAE for evaluating the performance of the proposed model.

## 4. Experimental Setup and Results

### 4.1. Assessment of ASS

To assess enterprises’ ASS, factor analysis was employed to extract the variables representing it. The features as shown in Table 2 were selected for EFA, which was shown by the results of the KMO test that the value of KMO was 0.813. Meanwhile, the results of Bartlett’s spherical test showed that the significant *p*-value was 0.000 *** (*** stands for 1% of the level of significance), which presented significance at the level, rejecting the null hypothesis that there was not a correlation between the variables and that the factor analysis was valid to the extent that it was suitable.

According to the EFA, to obtain the variables contained in each factor, the validation factor analysis was conducted. CFA requires that the total sample data should be at least five times the number of variables in the factor, and at least 200 samples were needed in general. In this experiment, with five factors and 18 variables, the dataset comprised 132,888 samples, meeting the basic requirements for CFA. According to the factor loading coefficients in Table 2, it could be seen that if the variables were within each factor (*p* were 0.000 ***) level of significance, then the original hypothesis was rejected, and it was considered that each factor loading was significantly different from zero. At the same time, its standardized loading coefficients were all greater than 0.6, which could be considered to have enough variance explained to show that each variable could be shown on the same factor.

According to Table 3 and Table 4, it could be seen that if the intra-factor aggregation validity was high and the square root of the AVE of the factor was greater than the Pearson correlation coefficient value of the other factors, then it showed that it had a more excellent discriminant validity.

The five variables in factor 2 were the number of the utilization rate of equipment statistics, the number of alarms, satellite positioning mileage, the number of vehicles, and the number of passes for dynamic data. The number of the utilization rate of equipment statistics and the number of passes for dynamic data reflected the enterprise’s emphasis on safety. Satellite positioning mileage and the number of vehicles reflected the enterprise’s scale. The number of alarms was related to traffic accidents, which was a more direct reflection of ASS. Therefore, compared with other variables within the factors, factor 2 better reflected the ASS of the enterprise, and factor 2 was chosen as the enterprise’s ASSI.

Based on the standardized loading coefficients corresponding to each variable in factor 2 and Equation (42), the daily ASSI of each road passenger transportation enterprise was obtained.

(41) $$y_{j}=\sum_{i=1}^{n}S_{ij}x_{ij}=0.843x_{1j}+0.910x_{2j}+0.905x_{3j}+0.865x_{4j}+0.898x_{5j}$$

(42) $$Y_{j}=\frac{y_{j}-y_{min}}{y_{max}-y_{min}}$$

where $Y_{j}$ is the ASSI of the jth enterprise, $S_{ij}$ is the standardized loading coefficient of the ith variable of the jth enterprise, $x_{i}$ is the value of the ith variable of the jth enterprise, n is the number of variables in factor 2, $x_{1j}$ is the number of the utilization rate of equipment statistics of the jth enterprise, $x_{2j}$ is the number of alarms in the jth enterprise, $x_{3j}$ is the satellite positioning mileage in the jth enterprise, $x_{4j}$ is the number of vehicles in the jth enterprise, $x_{5j}$ is the number of passes for dynamic data of the jth enterprise, and $y_{min}$, and $y_{max}$ are the minimum and maximum values in the ASSI of all enterprises, respectively.

**Figure 3:**
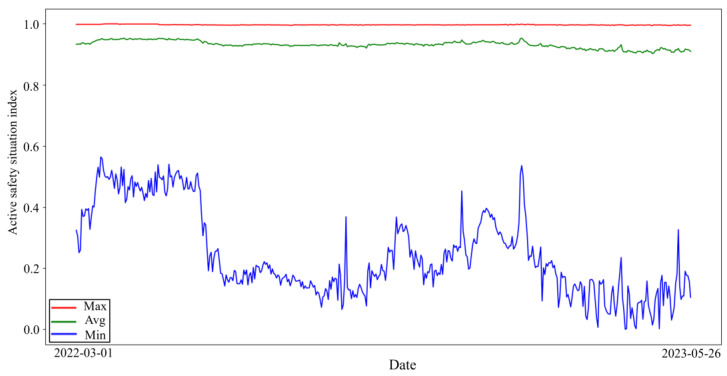
ASSI of enterprises.

**Figure 4:**
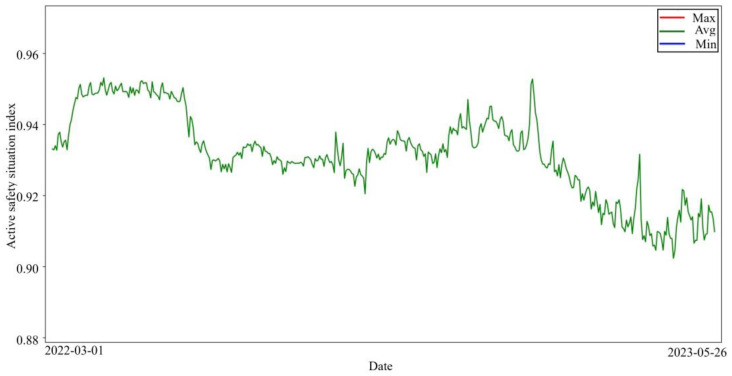
Zoom in on the average value of the enterprise ASSI.

The maximum, average, and minimum values of the ASSI of all enterprises for 452 days were plotted as shown in Figure 3. From Figure 3 and Figure 4, it can be seen that the maximum value of the daily enterprise ASSI fluctuates less. From the mean value, most of the enterprise ASSI is higher but the fluctuation trend is similar to the trend of the minimum value. The minimum value of the daily enterprise ASSI fluctuates in a wide range.

### 4.2. ASS Prediction

To compare the advantages of different time series prediction methods, this subsection selected the six models mentioned in Section 3.3 for the experiments, which were implemented via Python.

As for GRU, LSTM, Conv_LSTM, and TCN models, the selection of loss functions and optimizers is pivotal, given that the loss function gauges the disparity between predicted and actual values, thereby influencing model efficacy significantly [56]. Therefore, a trade-off was made among MSE, MAE, and SmoothL1 for the loss function, and among SGD, Adagrad, Adadelta, Adam, RMSprop, AdamW, and Nadam for the optimizer. ARIMA(p,d,q) automatically found the most appropriate p, d, and q for prediction based on the minimum AIC criterion through the auto_arima function in Python.

Prophet chose not to explicitly set N (the order of the seasonal pattern) and P (the period of the seasonality). The Prophet model in Python automatically detects seasonality in the data (based on the Fourier series) and models it accordingly, so there was usually no need to specify these parameters manually. Prophet tried to learn the period and pattern of the seasonality from the data and then performed an automatic fit.

Since taking the average value of all enterprises on a daily basis was considered as the ASS of the industry on that day, the dataset had a total of 452 days, in which each day’s data represented the safety situation of the whole industry. Considering the limited nature of the dataset, we chose to divide the dataset into a training set and a test set to better evaluate the generalization performance of the model. An 80:20 division ratio was used, where 80% of the data was used to train the model, while 20% was used to independently evaluate the performance of the model. This scientifically sound division helped ensure the effectiveness of the model in a wider range of contexts and improved generalization to future data.

Table 5 shows the test set optimization results for each model under each setup condition.

According to the results in Table 5, from the MSE point of view, Adam-TCN was better than the other five models in the experiment; from the MAE point of view, Adagrad-GRU was better than the other five models in the experiment.

### 4.3. Analysis of Factors Influencing ASS

The ASS of road passenger transportation enterprises selected relevant variables, including the number of calls received and made, number of physiological fatigue driving, number of fatigue driving, number of abnormal drivers, number of smoking, number of vehicles involved in speeding, number of night travel alarms, average speed while fatigued, utilization rate of equipment, total number of hours of alarms processed, and number of alarms handled. We input the selected relevant variables into the WDA-DBN model.

#### 4.3.1. Comparison of Methods

The proposed WDA-DBN model was utilized to explore the relationship between the filtered variables and ASS, as well as to compare the experimental effects before and after the algorithm improvement. Since there were a total of 132,888 data, for more data to be available for training and to improve the model’s generalization ability, 90% of the training dataset of about 119,599 was selected for training the model, and 10% of the data of about 13,289 was designated as the experimental set. For the experiments, epochs were selected as 50, 100, 150, and 200. In addition, to provide accurate prediction and enable the model to capture the association of features with ASS, we also selected two and three three layers of RBM and selected batches between 16, 32, and 64, where the number of output layers of two layers of RBM was 55 and 20, and the number of output layers of three layers of RBM was 55, 40, and 20, respectively. Epoch was not only set to 50, 100, 150, and 200 but also set to 100,000 for experiments for two-layer and three-layer DBN models.

We selected popular models which could select important variables as baseline models and chose common structural parameters for baseline models [57]. BPNN set the number of nodes in the hidden layer to 100 and the number of iterations to 50, 100, 150, and 200. XGBOOST set estimators to 100 and max depth to five.

Based on the best model derived from the experiments, the deep SHAP method was used to obtain the impact of each factor on ASS. Since the accurate estimation of the Shapley value might require a large amount of computation time, researchers usually select part of the data for calculating the Shapley value [58,59,60]. In this paper, we chose 500 samples from the test set for calculation.

Table 6 shows the test set optimization results for each model under each setup condition. The WDA-DBN was a three-layer RBM with batches 64 and epoch 100. The DBN was a two-layer RBM with epoch 100,000. XGBOOST setting was estimators 100 and max depth five. The number of nodes in the hidden layer of the BPNN was 100, which performed the same under four iteration counts.

According to the results summarized in Table 6, WDA-DBN showed the best performance compared to other models in terms of MSE and MAE.

#### 4.3.2. Influence Factor Analysis Based on DEEP SHAP

Based on the WDA-DBN using deep SHAP, the specific impact of each feature on ASS could be seen.

**Figure 5:**
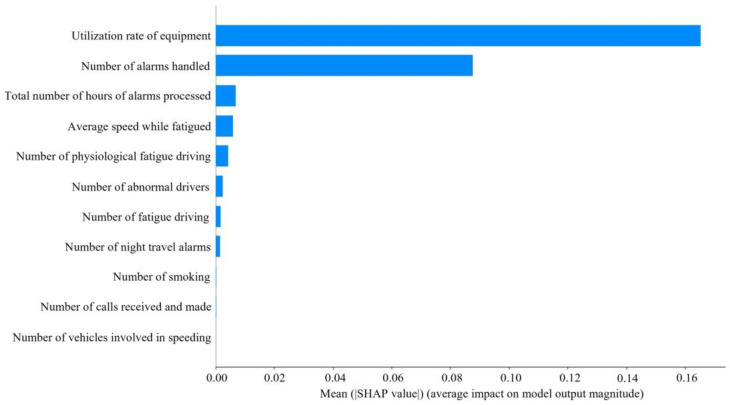
Variable contribution.

**Figure 6:**
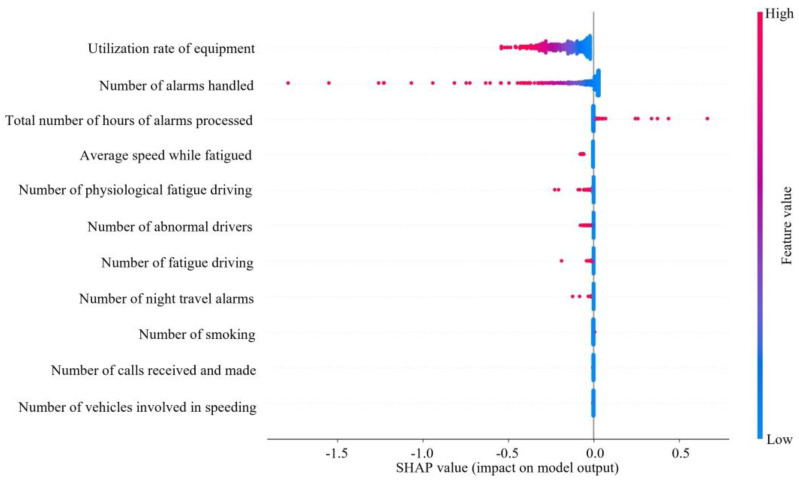
SHAP dependency analysis.

As can be seen in Figure 5 and Figure 6, the utilization rate of equipment had the greatest impact on ASS, followed by number of alarms handled. The higher the utilization rate of equipment, number of alarms handled, average speed while fatigued, number of physiological fatigue driving, number of fatigue driving, number of abnormal drivers, and number of night travel alarms processed, the lower the SHAP value and the lower ASS. The higher the value of the total number of hours of alarms processed, the higher the value of ASS. The number of smoking, number of calls received and made, and number of vehicles involved in speeding had a lesser effect on ASS.

## 5. Discussion

### 5.1. Assessment of ASS

From the results of Equation (40), the coefficient of the number of alarms in the enterprise’s ASS is the largest, followed by satellite positioning mileage. This indicates that the number of alarms is the most important part of the enterprise’s ASS. Enterprises and transportation authorities should strictly restrain the drivers in the enterprises and increase the supervision of the drivers so that the drivers can reduce the violations and thus improve the enterprises’ ASS. Satellite positioning mileage is the actual mileage of a day’s work of the driver in the enterprise, which reflects the size of the enterprise and the enterprise to the driver’s work intensity. At the same time, it is easy to think that the higher the satellite positioning mileage, the higher the number of alarms, and there is a correlation between the two variables. Therefore, the enterprise should be able to safely operate the enterprise with reasonable workload planning, and then determine the enterprise’s appropriate satellite positioning mileage.

According to the display in Figure 3, most enterprises have a high enterprise ASSI, which indicates that the ASS of the enterprise is relatively good. This situation is in line with common sense in the real world; as most of the enterprises have high safety awareness and adopt certain safety measures, the ASS of the enterprises is usually better. Observing the changing trend of the average of the enterprise’s ASSI can help management understand the average ASS of the industry so that management can implement measures to respond to possible industry challenges in a timely manner.

### 5.2. ASS Prediction

The improved model performance metrics, such as lower MSE and MAE, may be attributed to data cleaning and preprocessing, which reduces noise and enhances model accuracy. This allows for better detection of underlying patterns and dynamics in the time series data, resulting in reduced errors [61].

**Figure 7:**
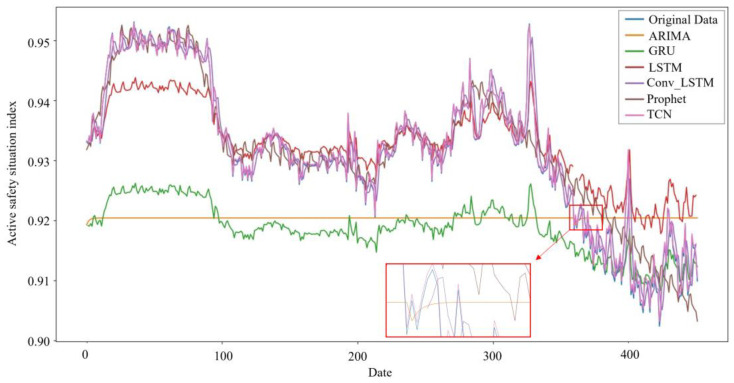
Time series model predictions.

According to the MSE of each model in Table 5, we can see that in terms of model performance, for predicting daily enterprise’s ASS, Adam-TCN, Adagrad-GRU, Adam-Conv_LSTM, Prophet, Adadelta-LSTM, and ARIMA(1,1,1) performance gradually decreased. From the perspective of MAE, it can be seen that the performance of Adagrad-GRU, Adam-TCN, Adam-Conv_LSTM, Prophet, Adadelta-LSTM, and ARIMA(1,1,1) gradually decreases. Generally, deep learning models outperform econometric models. Figure 7 illustrates that the ARIMA curve does not fit well with the original data curve, which may be because a simple econometric model makes it difficult to capture the complex dynamic characteristics of the data.

### 5.3. Relationship between ASS and Other Variables

Based on the results in Table 6, we can see that WDA-DBN is optimal and the 100 rounds of WDA-DBN outperform the 100,000 rounds of DBN by at least 7.66%. WDA-DBN is not only able to be more accurate than WDN but also to achieve similar performance when the number of rounds of operation should be greatly reduced to save the operating resources.

The utilization rate of equipment has the greatest impact on ASS, with the number of alarms handled coming in second. In fact, when a passenger driver is driving, the higher the utilization rate of equipment, the more dangerous driving behaviors will inevitably be detected. The number of alarms will increase, and ASS will decrease. The high number of alarms handled indicates that the driver has a good sense of active response, but it also indirectly indicates that number of alarms is high. Too many meaningless alarms will lead to increased operational risk. The processing of alarms requires frequent key presses, adjustments, or the replacement of settings. The operation is too complex and it may affect the driver’s emotions and responsiveness, which in turn affects safety.

The larger the value of total number of hours of alarms processed, the higher the value of ASS, which indicates that the positive response of the driver values safety, which is conducive to the improvement of the ASS of the enterprise. Calabrese et al. [62] similarly demonstrate the need for feedback.

The larger the value of average speed while fatigued, the lower the value of ASS. This is because when encountering danger, the faster speed of the vehicle requires the driver to have faster processing action. However, when the driver is fatigued, the driver’s concentration level and reaction time are reduced, making it difficult to avoid hazards in a timely manner. Borghetti, F. et al. [63] also found that among the most common causes of road accidents in driver behavior, high-speed driving is the most likely factor. Especially when fatigued, high-speed driving can further exacerbate unsafe conditions.

The number of physiological fatigue driving increases lead to ASS decreases. It may be because when the driver suffers from physiological fatigue, the driver will often relieve the discomfort caused by physiological fatigue through some simple activities, such as pressing the shoulder, arm, and waist with the hand. At this time, the driver’s attention to the outside world decreases and the hand is removed from the steering wheel, making it difficult to deal with sudden dangers. Driving fatigue negatively impacts safety, aligning with findings from previous studies [64].

The increase in the number of fatigue driving leads to a decrease in ASS, which is because the psychological pressure caused by drivers working for a long time increases, thus affecting safe driving. Cendales et al. [65] similarly argued that fatigue caused drivers to engage in dangerous driving behaviors.

The increase in the number of abnormal drivers leading to the decrease in ASS is due to the fact that drivers may lead to impulsive or irrational behaviors due to emotional fluctuations, such as anger, anxiety, and other abnormal emotions during driving. Lu et al. [66] similarly found that anger caused drivers to reduce their perception of risk, which in turn could affect driving safety.

The increase in number of night travel alarms leads to the decrease in ASS. It is due to the fact that visibility is poorer at night than during the daytime, which can make driving more difficult and make it more difficult for drivers to be aware of obstacles, other vehicles, and pedestrians on the road. Moreover, nighttime is the rest time of the body’s natural physiological cycle, and drivers are more likely to feel fatigued during this time. Fatigued driving reduces concentration and reaction time, and increases traffic risk. Lee et al. [35] similarly argued that night shift work increased driver drowsiness, reduced driving performance, and increased the risk of near-collision driving incidents.

Smoking, making and receiving calls, and speeding involving the number of vehicles have a few effects on ASS. The few effects of smoking on safety differed from previous studies in that Crizzle et al. [67] found that smoking caused crashes. The finding that talking on the phone has a smaller effect on safety is similar to Farmer et al. [68]. Farmer et al. [68] found that although an increase in cell phone use while driving should lead to an increase in crash rates, which had been decreasing in the years when cell phone use had been on the rise, crash rates were expected to increase significantly. The likelihood is that the increased risk of cell phone use and crashes due to cell phone use has been overestimated. It may also be that cell phone use has replaced other equally dangerous driving distractions. In this study, it could be because enterprises are stringent in managing violations such as smoking, making and answering phone calls, and speeding, which is one of the top five risk factors in the Global Status Report on Road Safety 2023. China enacted the Road Traffic Safety Law of the People’s Republic of China and the Regulations for the Implementation of the Road Traffic Safety Law of the People’s Republic of China to curb the occurrence of these three types of risky behaviors. These penalties have a greater deterrent effect, resulting in these violations occurring relatively infrequently, which in turn has less of an impact on an enterprise’s ASS. Sohaee, N. and Bohluli, S. [69] also proposed that policymakers should establish a comprehensive and multi-level regulatory framework and stricter enforcement of traffic safety laws and regulations, which would be beneficial for traffic safety. In addition, in real-world active safety posture systems for road transportation enterprises, there are often factors and noise or interference in the information that are difficult for humans to perceive, leading to the distortion of factor information, which differs from some research studies.

Enterprises and transportation authorities can use these results to formulate relevant management policies, such as requiring drivers to deal with alarm feedback when they receive it, finding a suitable place to take a break as soon as possible when drivers feel fatigued or are emotionally unstable, and prohibiting speeding and nighttime driving. By formulating management policies to restrain enterprises and drivers, the safety of long-distance passenger transportation will be improved, public safety will be protected, and property losses will be reduced.

## 6. Conclusions and Future Research

This study delves into the issue of assessing and predicting the ASS of road passenger transportation enterprises, with road passenger transportation enterprises of a province in China, as the object of study.

Through the EFA and CFA of individual characteristics, we successfully assessed the ASS of the enterprises and reliably predicted the ASS of the industry using a time series model. Our study found that among the models compared, the TCN model performed well on the MSE evaluation metrics, while the GRU model possessed a significant advantage on the MAE evaluation metrics. By assessing and predicting ASS, enterprises and transportation authorities can better understand the ASS of enterprises and industries, which provides strong support for enterprises and transportation authorities to propose decisions. Moreover, by TCN and GRU, enterprises and transportation authorities can foresee the development trend of ASS in advance, so as to provide a time window for strategic planning and adjust the existing resource allocation.

We introduce the WDA to optimize the DBN model and utilize DEEPSHAP to interpret this black-box model, effectively eliminating redundant information and uncovering factors with higher information content. We find that the total number of hours of alarms processed has a positive effect on ASS, while some other variables such as the number of fatigue driving, the number of abnormal drivers, and the number of night travel alarms have a negative effect on ASS. Variables such as the number of smoking, number of calls received and made, and number of vehicles involved in speeding have less impact on ASS. This provides transportation authorities and transportation enterprises with more operational information so that they can better understand and improve their safety management strategies. By effectively leveraging and processing information, this assists enterprises and transportation authorities in gaining earlier and better insights into the active safety posture of enterprises, and subsequently taking timely measures.

We not only present findings on how to synthesize an enterprise’s ASS and anticipate future trends in advance but also explore the mechanisms by which each variable influences ASS. These findings provide useful policy recommendations for transportation authorities and transportation enterprises to improve the ASS of road passenger transportation.

We acknowledge the following limitations in our study. Our predictive research is confined solely to common single time series forecasting models. To further expand the breadth and depth of our research, and to identify models more suitable for predicting the ASS of road transportation enterprises, it is necessary for us to introduce a greater variety of time series models and explore their combinations. Such exploration holds promise for providing us with more comprehensive and accurate prediction results, aiding enterprises in better understanding and addressing potential safety challenges, and further enhancing active safety management. Secondly, due to limitations in data collection, factors such as the completion rate of driver safety training, response rate to safety alerts, driver physiological characteristics, operational routes, driver aggressive behavior, and hours of non-compliance with mandatory rest periods were not considered. It is imperative for enterprises and transportation authorities to prioritize these factors and incorporate them into regulatory frameworks. In future work, we will rely on a nationally funded project to collect extensive data from locations such as Chongqing and An-hui in China, exploring the impact of the aforementioned factors on the ASS of urban public transportation, long-distance passenger transportation, and hazardous goods transportation enterprises, as well as their interrelationships. Considering these factors comprehensively will not only help improve model performance but also unearth more potential hazards affecting the enterprises’ ASS, assisting enterprises and transportation authorities in taking targeted measures to enhance enterprise safety. Lastly, our research findings indicate that variables such as smoking, phone use while driving, and speeding involving the number of vehicles have a minor impact on ASS, possibly influenced by policies, thereby affecting the reliability and generalizability of the study. In future research endeavors, we will gradually address these shortcomings.

## Figures and Tables

**Table 1:** Variables of the road transportation enterprises dataset.

Name	Description
Date	yyyy-mm-dd
Enterprise ID	The name of the enterprise
Area ID	The name of the area where the enterprise is located
Number of vehicles	The number of vehicles operated by an enterprise
Number of alarms	The total number of alarms for vehicles in the enterprise in one day
Satellite positioning mileage	Total miles traveled in one day by vehicles in the enterprise
Sum of severe alarm levels	The sum of serious alarm levels for vehicles in the enterprise in one day
Number of physiological fatigue driving	The total number of alarms due to physiological fatigue of drivers in the enterprise
Number of calls received and made	The total number of alarms caused by drivers in the business due to answering a phone call
Number of smoking	The total number of alarms due to smoking by drivers in the enterprise
Number of abnormal drivers	The total number of alarms caused by drivers in the enterprise due to abnormal emotions
Number of speeding	The total number of alarms due to speeding by drivers in the enterprise
Number of fatigue driving	The total number of alarms due to fatigued drivers in the enterprise
Number of night travel alarms	The total number of alarms caused by drivers in the enterprise due to driving at night during prohibited driving hours
Total number of violations of the night travel ban	The total number of times drivers in the enterprise drove at night during prohibited driving hours
Number of vehicles involved in the night travel ban	The number of vehicles in the enterprise that engage in driving behavior during hours when driving is prohibited at night
Cumulative hours of prohibited traffic violations	Cumulative total hours driven by drivers in the business during hours when driving is prohibited at night
Accumulated mileage violation of prohibited traffic	Total cumulative miles driven by drivers in the business during hours when driving at night is prohibited
Number of vehicles involved in speeding	The number of vehicles with speeding behavior in the enterprise
Accumulated hours of sustained speeding	The total cumulative hours of speeding by drivers in the business
Average speed while fatigued	The average speed at which drivers in the enterprise drive when fatigued
Number of vehicles involved in fatigued driving	The number of vehicles with fatigued driving behavior in the enterprise
Fatigue duration of driving	The total cumulative hours of driver fatigue driving in the enterprise
Utilization rate of equipment	The number of vehicles using onboard monitoring equipment in the enterprise / the total number of vehicles traveling in the enterprise on the same day
Number of utilization rate of equipment statistics	The number of times an enterprise counts the use of equipment in a day
Number of passes for dynamic data	The number of qualified times enterprise dynamic data is uploaded to the platform
Average alarm response time	The average length of time for drivers in the enterprise to respond after an alarm occurs
Total number of hours of alarms processed	The total time it takes for the driver in the enterprise to deal with the alarm after it occurs
Number of alarms handled	The total number of times drivers in the enterprise responded to alarms after they occurred

**Table 2:** Factor loading coefficients for road passenger enterprises.

Factor	Variable	Non-Standard Load Factors	Standardized Load Factor	z	S.E.	P
factor 1	The total number of violations of the nighttime ban	1.000	0.999	-	-	-
	The accumulated mileage violation of prohibited traffic	0.506	0.998	4995.594	0.000	0.000 ***
	The number of nighttime trip alarms	0.934	0.999	5564.666	0.000	0.000 ***
	The number of vehicles involved in the nighttime ban	0.933	0.999	5568.836	0.000	0.000 ***
	The cumulative hours of prohibited traffic violations	0.507	0.998	5144.007	0.000	0.000 ***
factor 2	The number of the utilization rate of equipment statistics	1.000	0.843	-	-	-
	The number of alarms	0.408	0.910	448.593	0.001	0.000 ***
	Satellite positioning mileage	0.411	0.905	444.678	0.001	0.000 ***
	Number of vehicles	0.524	0.865	409.552	0.001	0.000 ***
	The number of passes for dynamic data	0.411	0.898	438.003	0.001	0.000 ***
factor 3	The accumulated hours of sustained speeding	1.000	1.000	-	-	-
	The number of speeding	0.902	1.000	10,076.52	0.000	0.000 ***
	The umber of vehicles involved in speeding	1.039	0.999	9015.846	0.000	0.000 ***
factor 4	The fatigue duration of driving	1.000	0.999	-	-	-
	Average speed while fatigued	1.711	0.926	891.755	0.002	0.000 ***
	The number of vehicles involved in fatigued driving	0.823	0.999	6359.075	0.000	0.000 ***
	The number of fatigue driving	0.407	0.999	5569.513	0.000	0.000 ***
factor 5	The sum of severe alarm levels	1.000	1.000	-	-	-

**Table 5:** Comparison of predictive performance of different models.

Model	Loss Function	MSE	MAE
Adagrad-GRU	MAE	1.559 × 10^−6^	1.286 × 10^−4^
Adadelta-LSTM	MAE	1.092 × 10^−4^	9.753 × 10^−3^
ARIMA(1,1,1)	-	7.403 × 10^−4^	7.388 × 10^−3^
Prophet	-	4. 457 × 10^−5^	5.898 × 10^−3^
Adam-Conv_LSTM	MSE	1.989 × 10^−5^	3.511 × 10^−3^
Adam-TCN	MSE	2.944 × 10^−7^	5.333 × 10^−4^

**Table 6:** Comparison of performance metrics for various configurations of DBN models.

Model	MSE	MAE
WDA-DBN	0.0011975	0.0221929
DBN	0.0012969	0.0232784
XGBOOST	0.0012857	0.0221388
BPNN	0.0016549	0.02545431

**Table 3:** Results of the AVE and CR indicators for the road passenger transportation enterprise factor model.

Factor	AVE	CR
factor 1	0.998	0.999
factor 2	0.744	0.925
factor 3	0.999	1.000
factor 4	0.907	0.970
factor 5	0.999	0.999

**Table 4:** Results of the differentiation effect of the factor model for road passenger transportation enterprises.

	Factor 1	Factor 2	Factor 3	Factor 4	Factor 5
factor 1	0.999				
factor 2	0.092	0.863			
factor 3	0.300	0.112	0.999		
factor 4	0.157	0.185	0.227	0.952	
factor 5	0.334	0.126	0.614	0.710	0.999

## Data Availability

The data presented in this study are available upon request from the corresponding author. The data are not publicly available due to privacy.
